# Gene Correction of Point Mutations Using PolyPurine Reverse Hoogsteen Hairpins Technology

**DOI:** 10.3389/fgeed.2020.583577

**Published:** 2020-10-29

**Authors:** Alex J. Félix, Anna Solé, Véronique Noé, Carlos J. Ciudad

**Affiliations:** Department of Biochemistry and Physiology, School of Pharmacy and Food Sciences, and Institute for Nanoscience and Nanotechnology (IN2UB), University of Barcelona, Barcelona, Spain

**Keywords:** gene-editing, repair-PPRH, triplex, APRT, DHFR, mutation

## Abstract

Monogenic disorders are often the result of single point mutations in specific genes, leading to the production of non-functional proteins. Different blood disorders such as ß-thalassemia, sickle cell disease, hereditary spherocytosis, Fanconi anemia, and Hemophilia A and B are usually caused by point mutations. Gene editing tools including TALENs, ZFNs, or CRISPR/Cas platforms have been developed to correct mutations responsible for different diseases. However, alternative molecular tools such as triplex-forming oligonucleotides and their derivatives (e.g., peptide nucleic acids), not relying on nuclease activity, have also demonstrated their ability to correct mutations in the DNA. Here, we review the Repair-PolyPurine Reverse Hoogsteen hairpins (PPRHs) technology, which can represent an alternative gene editing tool within this field. Repair-PPRHs are non-modified single-stranded DNA molecules formed by two polypurine mirror repeat sequences linked by a five-thymidine bridge, followed by an extended sequence at one end of the molecule which is homologous to the DNA sequence to be repaired but containing the corrected nucleotide. The two polypurine arms of the PPRH are bound by intramolecular reverse-Hoogsteen bonds between the purines, thus forming a hairpin structure. This hairpin core binds to polypyrimidine tracts located relatively near the target mutation in the dsDNA in a sequence-specific manner by Watson-Crick bonds, thus producing a triplex structure which stimulates recombination. This technology has been successfully employed to repair a collection of mutants of the *dhfr* and *aprt* genes within their endogenous *loci* in mammalian cells and could be suitable for the correction of mutations responsible for blood disorders.

Scientists estimate that the global prevalence of all monogenic diseases in the human population is 1%, including over 10,000 different conditions (Control of hereditary diseases. Report of a WHO Scientific Group, 1996). These disorders are often the result of a unique single point mutation in a specific gene that produces a non-functional protein. Recently, nuclease-based gene editing tools such as transcription activator like nucleases, zinc-finger nucleases, or CRISPR/Cas platforms have been extensively used to correct mutations in the DNA (Gaj et al., 2016). Alternatively, molecules such as triplex-forming oligonucleotides (TFOs) (Seidman and Glazer, 2003) or peptide nucleic acids (PNAs) (Ricciardi et al., 2018b) that do not rely on the activity of nucleases to produce the gene correction have been developed. In this instance, the repair event is triggered by the formation of a local triple helix structure near the mutation site that stimulates the cell's own endogenous repair machinery. Here, we will review an alternative triplex-forming molecule named PolyPurine Reverse Hoogsteen (PPRH) hairpin, which has been developed in our laboratory, to correct point mutations in the DNA.

## PPRHs

PPRHs are non-modified single-stranded DNA molecules (45–55 nt) formed by two polypurine mirror repeat sequences linked by a five-thymidine bridge (5T). The formation of the hairpin structure is due to the establishment of intramolecular reverse-Hoogsteen bonds between the purines. PPRHs can bind to polypyrimidine tracts in the double-stranded DNA (dsDNA) in a sequence-specific manner via Watson-Crick bonds, thus generating a triple helix in the target site and displacing the polypurine strand of the dsDNA (Coma et al., 2005). This local distortion in the dsDNA interferes with DNA transcription and inhibits the expression of the targeted gene (de Almagro et al., 2009).

During the last decade, we have used PPRHs as gene silencing tools to inhibit genes related to cancer progression such as *dihydrofolate reductase* (*DHFR*) (de Almagro et al., 2009, 2011), *telomerase (TERT)* (de Almagro et al., 2009), *BCL2, topoisomerase 1* (*TOP1), mTOR, MDM2*, C-*MYC* (Villalobos et al., 2015)*, CHK1, WEE1* (Aubets et al., 2020) and s*urvivin* (*BIRC5*) *in vivo* (Rodríguez et al., 2013). Additionally, we applied the PPRHs technology in immunotherapy approaches by inhibiting the CD47/SIRPα (Bener et al., 2016) and PD-1/PD-L1 pathways (Enríquez et al., 2018; Ciudad et al., 2019). PPRHs and their advantages (low cost of production, stability, and lack of immunogenicity) as gene silencing tools for cancer have been reviewed in Ciudad et al. (2017).

## Repair-PPRHs

**Figure 1:**
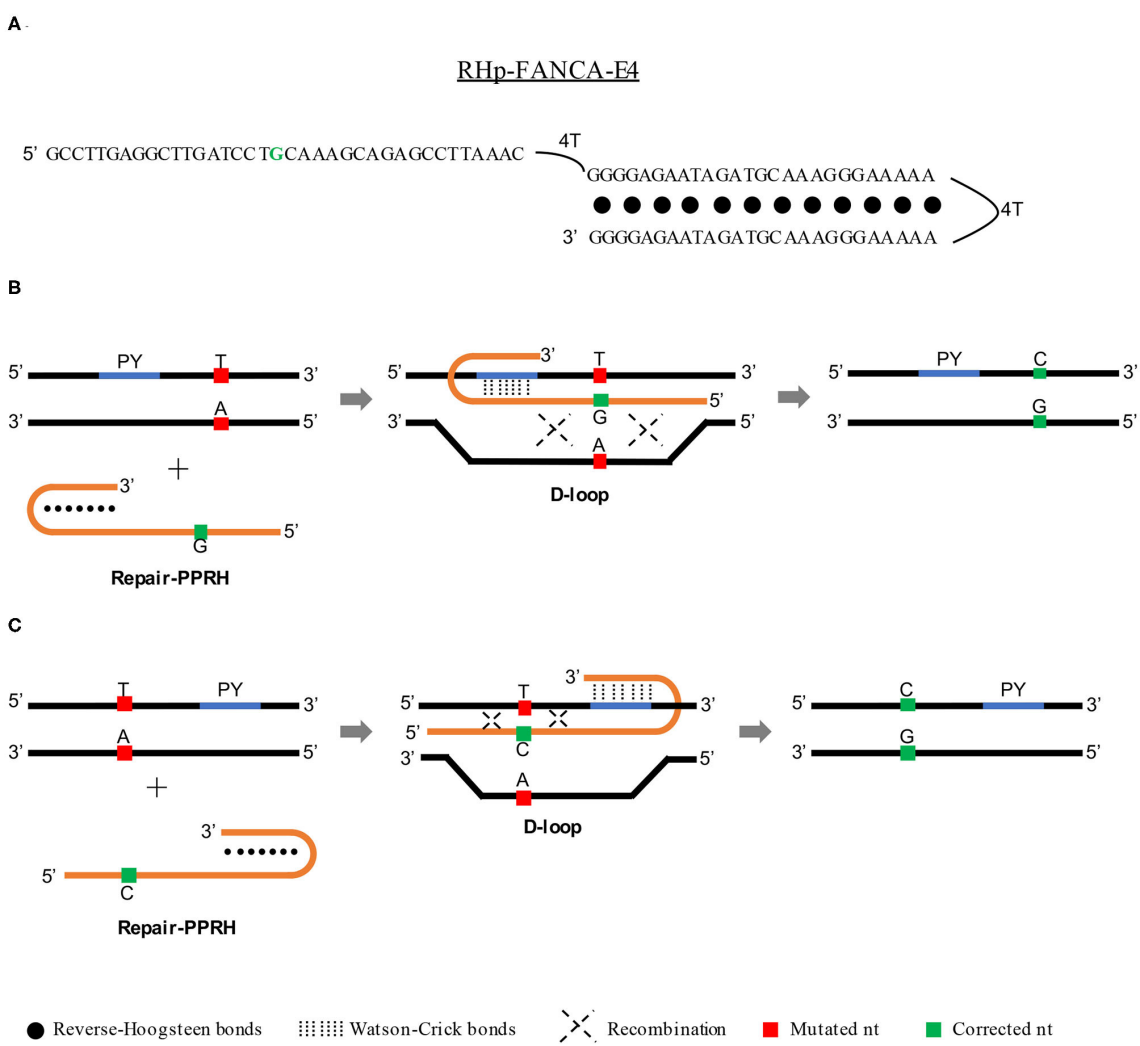
Mechanism of action of repair-PPRHs. **(A)** Representation of the RHp-FANCA-E4 repair-PPRH targeting the c.295 C>T mutation in the *FANCA* gene. In this case, the polypurine hairpin core is bound to the repair domain by an additional four-thymidine bridge following the long-distance repair-PPRH approach. Scheme depicting the mechanism of action of a repair-PPRH when the polypyrimidine target sequence (PY) is located either upstream **(B)** or downstream **(C)** of the mutation.

It is known that triplex formation can stimulate repair between a targeted *locus* and a donor DNA sequence by both homology-directed repair (HDR) (Datta et al., 2001; Knauert et al., 2006) and nucleotide excision repair (NER) (Faruqi et al., 2000; Datta et al., 2001; Rogers et al., 2002) pathways. For that reason, we believed that PPRHs could represent an alternative tool for gene correction due to their ability to produce triplex structures and therefore stimulate recombination (between the template and the target site) to correct point mutations in the DNA. To do so, we conceived an advanced design of the PPRH molecules that we called repair-PPRHs. These molecules are PPRH hairpins that bear an extension sequence at one end of the molecule which is homologous to the DNA sequence to be repaired but including the corrected nucleotide instead of the mutated one (Figure 1A). In this case, the polypurine hairpin core of the repair-PPRH is designed to bind to a polypyrimidine sequence located near the target mutation, thus producing the PPRH/DNA triplex and stimulating the recombination between the extension sequence of the repair-PPRH and the mutation target site.

In our seminal paper we used repair-PPRHs to correct a point mutation in the *dhfr* gene from Chinese Hamster Ovary (CHO) cells (Solé et al., 2014). We selected the *dhfr* gene as a model because we could easily identify the repaired clones by applying a DHFR selective culture medium that does not contain glycine, hypoxanthine nor thymidine (-GHT).

First, DNA binding assays were performed to check the capacity of PPRHs to open the target dsDNA for the subsequent binding of a repair oligonucleotide corresponding to the extension sequence of the repair-PPRH. Two PPRHs containing 13 and 23 purines, respectively, directed against polypyrimidine sequences located in exon 6 of the *dhfr* gene were used to perform the binding experiments. We demonstrated that both PPRHs were able to bind and open their target dsDNA sequences ranging from 13 to 25 nt. Moreover, the introduction of an interruption in the duplex to simulate a point mutation did not alter the binding of the PPRH to its target sequence (Solé et al., 2014). The minimum concentration to obtain the binding between the PPRH and its target sequence was 3 nM. Additionally, (Solé et al., 2017) proved that even PPRHs susceptible to fold into stable G4 structures can still bind in a sequence-specific manner to the target DNA and produce triplex formation.

Then, to assess if PPRHs were able to correct a point mutation, we designed a repair-PPRH directed against a non-sense mutation (G>C) located in exon 2 of the *dhfr* minigene contained in the p11Mut expression vector. To do so, a PPRH bearing a polypurine hairpin core of 13 nt was combined with a 25 nt extension sequence homologous to the mutation site but containing the corrected nucleotide. In cells, two different approaches were attempted to repair this mutation in p11Mut. In the first approach, gene correction was achieved by the co-transfection of both p11Mut and the repair-PPRH in *dhfr*-deficient DG44 CHO cells. After incubation, cells were selected in -GHT medium obtaining different repaired clones. The frequency of repair was ~0.15% (Solé et al., 2014). Gene correction was confirmed by DNA sequencing and by determining the levels of DHFR mRNA and protein. In the second approach, we performed the experiment in DG44 cells stably transfected with p11Mut (DG44-p11Mut cell line) since it could resemble to our final aim of correcting a point mutation in the endogenous *locus* of the gene. We confirmed that the repair-PPRH was able to correct the mutation at the same frequency (0.15%) as our first approach (Solé et al., 2014). The levels of DHFR mRNA and protein were recovered compared to the mutant DG44-p11Mut cell line (Solé et al., 2014). In a third approach, we explored the applicability of repair-PPRHs to correct point mutations at the endogenous level. There, a repair-PPRH designed against a mutation in exon 6 (G>-) of the *dhfr* gene was transfected into the DA5 cell line, which contained this specific mutation in the endogenous *locus* of the *dhfr* gene. After selection, surviving cell colonies were acquired at a frequency of 0.01% (Solé et al., 2014). In this case, gene correction frequency was lower than in the previous experiments since the correction was achieved for the first time in the endogenous *locus* of the gene. However, spontaneous corrections were not observed in any of the experiments. The levels of DHFR mRNA and protein were rescued compared to the mutant DA5 cell line. Moreover, we corroborated that the DHFR protein from the repaired clones showed equal or higher DHFR activity levels than the *dhfr*^+^ parental cell line, thus demonstrating that the corrected gene was completely functional (Solé et al., 2014).

## Factors Affecting Gene Correction Frequency

The study of the influence of both hydroxyurea and aphidicolin in the repair frequency was also addressed (Solé et al., 2014). It is known that hydroxyurea inhibits the ribonucleotide reductase enzyme (Bianchi et al., 1986), thus arresting cells in the S phase of the cell cycle by blocking or retarding the movement of the replication fork caused by the dNTP pools imbalance (Saintigny et al., 2001). In the case of aphidicolin, it is a potent inhibitor of polymerases α, δ and ε, which leads to the blockage of the replication fork and provokes a similar effect to hydroxyurea (Wang, 1991). The effect on replication caused by these agents leads to double-strand DNA breaks (DSBs), which can stimulate both the HDR and the non-homologous end joining (NHEJ) pathways to repair the DNA damage (Lundin et al., 2002). Accordingly, the incubation of both DG44 and DG44-p11Mut cell lines with 5 μg/mL aphidicolin or 2 mM hydroxyurea for 3 h before incubation with the repair-PPRHs increased the repair frequency by 2-fold (Solé et al., 2014). This is in keeping with other studies showing increased gene correction frequencies when incubating repair oligonucleotides after treatment with hydroxyurea or aphidicolin (Parekh-Olmedo et al., 2003; Ferrara et al., 2004; Wu et al., 2005; Chin et al., 2008; Engstrom and Kmiec, 2008).

Finally, since the RAD51 protein plays a central role in homologous recombination (Krejci et al., 2012; Papaioannou et al., 2012) and it is required for triplex-induced recombination (Datta et al., 2001; Gupta et al., 2002), we checked its role in the repair event triggered by repair-PPRHs. Co-transfection of the repair-PPRH with a pRad51 expression vector in DA5 cells led to an increase in gene correction frequency of 10-fold compared to the transfection of the repair-PPRH alone (Solé et al., 2014), thus confirming that homologous recombination is involved in the repair process. Overall, this study represented the proof-of-concept for the usage of PPRHs as gene editing tools.

## Correction of Point Mutations in the Endogenous *Locus*

In the following study, the usage of repair-PPRHs was expanded by correcting a representative compilation of point mutations (insertions, deletions, substitutions, and a double substitution) located in the endogenous *locus* of the *dhfr* gene (Solé et al., 2016). For that purpose, *dhfr*-deficient CHO cell lines derived from the parental cell line UA21 (Urlaub et al., 1983), which carried only one copy of the *dhfr* gene (hemizygous), were selected to perform the repair experiments. DU8 (Urlaub et al., 1989), DF42 (Carothers et al., 1986), DI33A (Chasin et al., 1990; Carothers et al., 1993a), DA5 and DA7 (Carothers et al., 1993b) and DP12B and DP6B (Carothers et al., 1993a) cell lines contained premature STOP codons either in place by a nucleotide substitution or downstream due to frameshift by single deletions, insertions, or by exon skipping, thus producing a non-functional DHFR enzyme (Table 1). Repair-PRHs were designed targeting the different mutations and transfected in their corresponding mutant cell lines. After selection in -GHT deficient medium, repaired clones were expanded and analyzed by DNA sequencing of the targeted site, thus demonstrating the correction of the mutation. We also confirmed that the corrected *dhfr* gene was completely functional since the levels of DHFR mRNA and protein were equal or higher than the levels shown by the parental cell line, as well as DHFR enzymatic activity (Solé et al., 2016). In addition, we evaluated the variation in gene correction frequency depending on the number of DF42 cells initially plated to perform the experiment. The maximum frequency value was observed (7.6%) when transfection was carried out with only 1,000 cells (Solé et al., 2016).

**Table 1:** CHO mutant cell lines corrected by repair-PPRHs.

**Cell line**	**Gene**	**Mutation**	**Base change**	**Coding change**	**References**
DF42	*dhfr*	c.541 Exon 6	Substitution G > T	STOP in place	Solé et al., 2016
DA5		c. 541 Exon 6	Deletion (-G)	STOP at +584 (normal termination is at +562)	
DP12B		c.370 – 2 Intron 4	Substitution A > T	Exon 5 skipped STOP at +504	
DI33A		c. 493 Exon 6	Insertion (+G)	STOP at +505	
DU8		c. 136 + 1 Exon 2/Intron 2	Double substitution GG > AA	Exon 2 skipped STOP at +139	
DA7		c. 235 Exon 3	Substitution G > T	STOP in place	
S23	*aprt*	c. 7 Exon 1	Substitution G > T	STOP in place	Félix et al., 2020
S1		c. 180 Exon 2	Substitution C > G	STOP in place	
S62		c. 505 Exon 5	Substitution G > T	STOP in place	

*Position numbers refer to the translational start site (ATG). The correction of the mutant cell lines using repair-PPRHs can be found in the referenced papers*.

One can argue that PPRH molecules present a major limitation since it is necessary to find polypyrimidine stretches relatively close to the target mutation. Despite these polypyrimidine domains are more abundant in the human genome than initially predicted by simple random models (Goñi et al., 2004, 2006), finding a polypyrimidine sequence adjacent to the point mutation can be complicated in some cases. To solve this issue for the DF42 mutant, we designed a long-distance repair-PPRH whose repair domain was targeting the mutation located 662 nt upstream from the polypyrimidine target sequence of the hairpin core. The repair domain of the repair-PPRH was connected to the hairpin core by another 5T loop. This long-distance repair-PPRH was able to correct its targeted mutation showing similar results to the short-distance repair-PPRH used for the correction of the same mutant, thus indicating that adjacency between the target mutation and the polypyrimidine domain was not crucial to achieve the correction.

## Generality of Action of Repair-PPRHs

Recently, we demonstrated the generality of action of repair-PPRHs (Félix et al., 2020) by correcting three different mutations in the endogenous *locus* of the *aprt* gene in various *aprt-*deficient CHO cell lines (Table 1) named S23, S62, and S1 (Phear et al., 1989). It is worth noting that this gene also served as a disease model in CHO cells, since *aprt* deficiency in humans represents an inherited condition that severely affects the urinary tract and the kidneys (Bollée et al., 2012; Edvardsson et al., 2019). In that study, we designed repair-PPRHs containing polypurine hairpin cores composed of 19–22 nt to assure their specificity and to minimize the off-target effects as much as possible. In all the mutant cell lines we demonstrated the correction of the mutation at the DNA, mRNA and enzymatic levels, showing that the corrected APRT protein was completely functional. Moreover, we used a long-distance repair-PPRH in which the polypyrimidine target sequence was located 24 nt downstream of the S1 mutation site, however, it showed a similar effect to that of the short-distance repair-PPRH (Félix et al., 2020). The influence of the cell cycle phase in the repair event was also studied by performing gene correction experiments either during S phase or in asynchronous conditions. The repair frequency was increased by 2.5-fold in S phase (Félix et al., 2020), which is in accordance with other studies regarding gene correction with repair oligonucleotides (Majumdar et al., 2003; Brachman and Kmiec, 2005; Olsen et al., 2005).

One of our concerns was the possible generation of off-target edits in the repaired genome caused by the treatment with repair-PPRHs. Whole genome sequencing analyses of repaired clones revealed that the repair-PPRH did not produce any random insertions or deletions (indels) in the genome. Moreover, the sequence of the repair-PPRH itself was not detected in any location of the genome (Félix et al., 2020). Finally, we got an insight into the molecular mechanism responsible for the gene correction event. The D-loop structure formation upon binding of the repair-PPRH to its polypyrimidine target sequence was demonstrated by DNA binding assays (Félix et al., 2020), thus serving as a recombination intermediate that stimulates DNA repair (Parekh-Olmedo et al., 2002; Drury and Kmiec, 2003, 2004). The mechanism of action of repair-PPRHs is depicted in Figures 1B,C.

Despite the advantages of repair-PPRHs, we would like to state that the main limitations of this technology are the low repair frequency and the delivery. A way to ameliorate the low repair frequency would be to increase the rate of homologous recombination. In this direction, as stated previously, co-transfection of repair-PPRHs with a pRAD51 led to an increase in the correction frequency. Since the rate of homologous recombination is higher in the S phase of the cell cycle, synchronization in the S phase can also increase the correction frequency, as observed for the *dhfr* and *aprt* genes. Regarding the delivery of repair-PPRHs, the development of new liposome formulations (Juliano, 2016) or polymeric nanoparticles (McNeer et al., 2015; Bahal et al., 2016; Ricciardi et al., 2018a) may contribute to improve gene repair. Finally, modification in the backbone of repair-PPRHs including phosphorothioate or locked nucleic acids (LNA) may increase the stability of the molecule and decrease its degradation by nucleases.

To date, we have only tested repair-PPRHs to correct single and double point mutations. Anyhow, most monogenic diseases are just caused by one point mutation in the responsible gene, thus making repair-PPRHs an alternative tool to correct different disorders. In this respect, we constructed Table 2 to show the versatility for designing repair-PPRHs to correct some of the most common point mutations that affect genes involved in monogenic blood disorders, with the aim of making them available for the scientific community.

**Table 2:** Compendium of repair-PPRHs designed to correct point mutations responsible for 10 different blood disorders.

**Blood disorder**	**Gene**	**Mutation**	**Codon change**	**Name and sequence (5^**′**^->3^**′**^) of the Repair-PPRH**
G6PD deficiency (mediterranean)	*G6PD*	c.563 C>T Exon 6	TCC>TTC Ser>Phe p.188	RHp-G6PD-E6-C (99 nt) GCCGTCACCAAGAACATTCACGAGTCCTGCATGAGCCAGATGTAAGGC TTGGGCAACGGGAGGGAAGGGCGGAttttAGGCGGGAAGGGAGGGCAACGG
Beta-Thalassemia	*HBB*	G>A Intron 1 (+110)	TGG>TAG	RHp-HBB-I1-C (91 nt) ACTGACTCTCTCTGCCTATTGGTCTATTTTCCCACCCTTAGttt tAAAAGAAAGGGGAAGAAAAGAttttAGAAAAGAAGGGGAAAGAAAA
Sickle cell disease	*HBB*	c.70 A>T Exon 1	GAG>GTG Glu>Val p.7	RHp-HBB-E-T (81 nt) CATGGTGCATCTGACTCCTGAGGAGAAGTCTGCCGTTACTGCCCTGT GGGGCAAGGTGAACGttttGCAAGTGGAACGGGG
Porphyria	*HMBS*	c.33+1 G>A/T Exon 1/ Intron 1	Intron retention 67 bp	RHp-HMBS-E1-T (97 nt) GCAATGCGGCTGCAACGGCGGTGAGTGCTGAGCCGGTGACCttt tGGAAGGAATGGGGAAATCAGAGAGttttGAGAGACTAAAGGGGTAAGGAAGG
Ferritin Deficiency	*FTL*	c.310 G>T Exon 3	GAG>TAG Glu>Ter p.104	RHp-FTL-E3-C (93 nt) TGAAAGCTGCCATGGCCCTGGAGAAAAAGCTGAACCAGGCCttt tGGAAAAGAGGGGGAGAGAGCAGttttGGAAAAGAGGGGGAGAGAGCAG
Dyserythropoietic anemia	*CODAN1B*	c.281 A>G Exon 5	TAT>TGT Tyr>Cys p.94	RHp-C15ORF41-E5-T (102 nt) GAGCCATTAATGAGGGCGCATAGTCCACCTCATTGGCCAGGTCCAGGAGCACTGGGG CAGGAGGTAAAAAGTGGTGAGGttttGGAGTGGTGAAAAATGGAG
Hemophilia A	*F8*	c.6976 C>T Exon 27	CGA>TGA Arg>Ter p.2326	RHp-F8-E27 (87 nt) CGTTACTGACTCGCTACCTTCGAATTCACCCCCAGAGTTGGtttt GGCAGTGGAGAGGGAGGAGttttGAGGAGGGAGAGGTGACGG
Hemophilia B	*F9*	c.169 C>T Exon 2	CAA>TAA Gln>Ter p.57	RHp-F9-E2 (100 nt) ATTCTCTCTCAAGGTTCCCTTGAACAAACTCTTCCAATTTACCTtttt AAGAAAAACTGAAATGTAAAAGAAttttAAGAAAATGTAAAGTCAAAAAGAA
Fanconi anemia	*FANCA*	c.295 C>T Exon 4	CAG>TAG Gln>Ter p.99	RHp-FANCA-E4 (99 nt) GCCTTGAGGCTTGATCCTGCAAAGCAGAGCCTTAAACtttt GGGGAGAATAGATGCAAAGGGAAAAAttttAAAAAGGGAAACGTAGATAAGAGGGG
Von Willebrand	*VWF*	c.4975 C>T Exon 28	CGA>TGA Arg>Ter p.1659	RHp-VWF-E28 (103 nt) GACGCTCCCCCGAGAGGCTCCTGACCTGGTGCTGCAGAGGTGCTGCTCCGGAGAGG GGCTGCAGAAGGGGTGGGAGAGGGGAttttAGGGGAGAGGGTGGGGA

*The design of the different repair-PPRHs was performed as follows: (i) Finding triplex targeting sites near the mutation using the TFO searching tool (http://utw10685.utweb.utexas.edu/tfo/) (Gaddis et al., 2006); (ii) Devising the corresponding polypurine hairpin core (underlined sequences); (iii) Determining the repair domain of the repair-PPRH corresponding to the homologous sequence of the mutation site but containing the corrected nucleotide (green). In the case of a long-distance repair-PPRH, an additional 4–5 thymidine loop is added between the hairpin core and the repair domain. The abbreviation of the gene responsible for the blood disorder, the position of the mutation and the affected codon are given for each case. The position of the mutation is referred to the translation start site (ATG). TER, termination codon*.

## CRISPR/Cas Systems

Nowadays, CRISPR/Cas has become a popular gene editing tool for therapeutic purposes (Osborn et al., 2015; Dever et al., 2016; Sansbury et al., 2019; van de Vrugt et al., 2019; Xiong et al., 2019). Nevertheless, several studies have demonstrated the presence of off-target effects caused by unspecific activity of the CRISPR/Cas system (Cradick et al., 2013; Lin et al., 2014; Schaefer et al., 2017; Anderson et al., 2018; Allen et al., 2019; Cullot et al., 2019). Unintended on-target effects such as large deletions and complex rearrangements have also been reported (Kosicki et al., 2018). In this regard, Félix et al. showed the absence of off-target effects when using repair-PPRHs to correct point mutations in the *aprt* gene in mammalian cells. Furthermore, since *Staphylococcus pyogenes* and *Staphylococcus aureus* cause infections at high frequencies in human beings, an anti-Cas9 preexisting effector T cell response has been discovered (Charlesworth et al., 2019; Wagner et al., 2019). On the other hand, PPRHs are non-modified (cheap) DNA oligonucleotides that do not activate the innate inflammatory response (Villalobos et al., 2014).

## TFOs

The ability of TFOs to stimulate recombination by triple helix formation in mammalian cells was first described in 1996 (Faruqi et al., 1996). Consecutive studies highlighted the potential of TFOs to correct mutations in the DNA by triplex-induced recombination between the target site and a donor DNA molecule (Chan et al., 1999; Culver et al., 1999; Datta et al., 2001). TFO backbone modifications have been developed to increase its binding affinity while reducing nuclease-mediated degradation. Peptide nucleic acids (PNAs) are synthetic DNA analogs composed of N-(2-aminoethyl)-glycine monomers linked by peptide bonds (Nielsen et al., 1991). This neutrally charged backbone allows the PNA to bind with high affinity to DNA, thus forming more stable triplex structures (Kim et al., 1993). Moreover, PNAs are also resistant to nuclease and protease activities (Demidov et al., 1994).

PNAs and their derivatives have been developed to correct mutations responsible for different monogenic diseases. Intranasal delivery of polymeric nanoparticles containing PNAs and donor DNA sequences in cystic fibrosis mice led to the correction of the F508del *CFTR* mutation *in vivo* (McNeer et al., 2015). More recently, PNAs delivered by polymeric nanoparticles have been used to correct the ß*-globin* gene both *in vivo* (Bahal et al., 2016) and *in utero* (Ricciardi et al., 2018a) in ß-thalassemic mice with very low off-target activity. The most recent review on PNAs as gene editing tools can be found in Economos et al. (2020).

## Final Remarks

It is evident that triplex-mediated repair of mutations in the DNA constitute a powerful gene editing approach that has demonstrated its therapeutic effect *in vivo*. Repair-PPRHs can represent a new tool in this field since they have shown their efficacy to correct different point mutations in the *dhfr* and *aprt loci* in mammalian cells with no detectable off-target activity. In addition, here we describe a collection of repair-PPRHs designed to correct 10 different blood diseases. A better understanding of the mechanisms by which the repair-PPRH triggers the recombination event may lead to improvements on PPRH design, thus increasing the frequency of correction.

## Author Contributions

AF, AS, and CC wrote the original draft and VN revised the manuscript. All authors contributed to the article and approved the submitted version.

## Conflict of Interest

The authors declare that the research was conducted in the absence of any commercial or financial relationships that could be construed as a potential conflict of interest.
